# The use of N-of-1 trials to generate real-world evidence for optimal treatment of individuals and populations

**DOI:** 10.1017/cts.2023.604

**Published:** 2023-09-06

**Authors:** Harry P. Selker, Dorothy Dulko, David J. Greenblatt, Marisha Palm, Ludovic Trinquart

**Affiliations:** 1 Institute for Clinical Research and Health Policy Studies (ICRHPS), Tufts Medical Center, Boston, MA, USA; 2 Tufts Clinical and Translational Science Institute (CTSI), Tufts University, Boston, MA, USA

**Keywords:** N-of-1 trial, real-world evidence, real-world data, personalized medicine, effectiveness trials, patient-centered research

## Abstract

**Introduction:**

Ideally, real-world data (RWD) collected to generate real-world evidence (RWE) should lead to impact on the care and health of real-world patients. Deriving from care in which clinicians and patients try various treatments to inform therapeutic decisions, N-of-1 trials bring scientific methods to real-world practice.

**Methods:**

These single-patient crossover trials generate RWD and RWE by giving individual patients various treatments in a double-blinded way in sequential periods to determine the most effective treatment for a given patient.

**Results:**

This approach is most often used for patients with chronic, relatively stable conditions that provide the opportunity to make comparisons over multiple treatment periods, termed Type 1 N-of-1 trials. These are most helpful when there is heterogeneity of treatment effects among patients and no a *priori* best option. N-of-1 trials also can be done for patients with rare diseases, potentially testing only one treatment, to generate evidence for personalized treatment decisions, designated as Type 2 N-of-1 trials. With both types, in addition to informing individual’s treatments, when uniform protocols are used for multiple patients with the same condition, the data collected in the individual N-of-1 trials can be aggregated to provide RWD/RWE to inform more general use of the treatments. Thereby, N-of-1 trials can provide RWE for the care of individuals and for populations.

**Conclusions:**

To fulfill this potential, we believe N-of-1 trials should be built into our current healthcare ecosystem. To this end, we are building the needed infrastructure and engaging the stakeholders who should receive value from this approach.

## Introduction

There has been an increasing understanding of the potential of real-world data (RWD) for generating real-world evidence (RWE) to inform clinical treatment. The potential utility of the many forms of data generated in clinical care via the use of electronic health records, devices, patient-reported outcomes, and other sources has led to a great interest in the use of RWD/RWE. This has been accelerated by the increasing acceptance of RWD/RWE by regulatory agencies. Although the Food and Drug Administration (FDA) initially emphasized RWD for post-market data collection, this approach also has been used as part of demonstrating efficacy for initial approval [1–2]. We believe that another source of RWD/RWE that will provide evidence for the care of individual patients and potentially of general efficacy is the use of N-of-1 trials.

In real-world care, clinicians and patients often try a series of treatments to determine which one works best for a patient, to inform long-term treatment. In usual clinical practice, treatment decisions may be directed by clinician judgment and preferences, anecdotal experience, and trial-and-error [3]. Even when high-quality evidence from randomized controlled trials (RCTs) is available to guide treatment, data from comparative effectiveness trials of the treatments being considered often are not available. Additionally, even with applicable trials, the evidence may not apply to a given patient. For example, many real-world patients do not meet the eligibility requirements or care settings used in the original pivotal efficacy RCTs for a given treatment. If these are importantly different from those of the reference RCTs, then the trial results may not apply. Moreover, although well-designed RCTs demonstrate average treatment effects for their samples, these average results will not apply to all individual real-world patients because of heterogeneity of treatment effects.

In this context, to personalize treatments, rather than turning to trial-and-error testing of different candidate treatments in an informal way, N-of-1 trials – sometimes referred to as single-patient or personalized trials – use rigorous processes to provide reliable evidence relevant to an individual real-world patient’s care. In N-of-1 trials, patients are given candidate treatments (which may include placebo), allocated in random order over a series of periods, during which neither the patient nor the clinician knows which treatment is being given (i.e., double-blinded). This combines real-world clinical practice with contemporary clinical research methods to determine the most effective treatment for that patient. Thereby, RWD and RWE are generated for individual patients in a way that is patient-centric and reinforces patient-physician shared decision-making about treatments [4]. When used to inform clinical decision-making, N-of-1 trials offer an approach that reduces the speculation that is intrinsic to usual clinical practice [3].

As N-of-1 trials apply concepts from RCT design, despite being conducted in small samples, they can generate high-level evidence [5]. The Oxford Centre for Evidence-Based Medicine includes N-of-1 trials as a high level-1 evidence, specifically when proposing a clinical hypothesis applied to an individual patient [6]. This level of evidence is aligned with that of an RCT, unlike individual case studies, which are considered a lower level of evidence (level 4), largely due to the risk of bias (e.g., associated with non-blinded treatments, non-randomized sequences of exposure, and expectations about favorable and unfavorable medication effects by patients and clinicians) that can affect outcomes.

Although potentially attractive for patients, to date, N-of-1 studies have not been widely used in usual clinical practice. Thus, patients do not benefit from personalized evidence and other parts of the healthcare ecosystem also do not get potential benefits. Believing that this is a missed opportunity, Tufts Clinical and Translational Science Institute (CTSI) has embarked on a project to widen the use of N-of-1 trials, first demonstrating the embedding of this approach across our health system, Tufts Medicine, starting with rheumatoid arthritis and asthma, and then helping to disseminate the approach and our learnings more widely.

## Implementation of N-of-1 Trials

Believing that wide use of N-of-1 trials should lead to benefits for patients and key members of the healthcare enterprise, we started by engaging all likely stakeholders to understand their perspectives of the potential benefits of embedding N-of-1 trials into the healthcare ecosystem [7]. Incorporating those viewpoints, we now are piloting this approach at Tufts Medicine with the goal of making it available for the care of real-world patients and for generating RWD/RWE on treatments for individual patients and for more general use.

**Figure 1.**
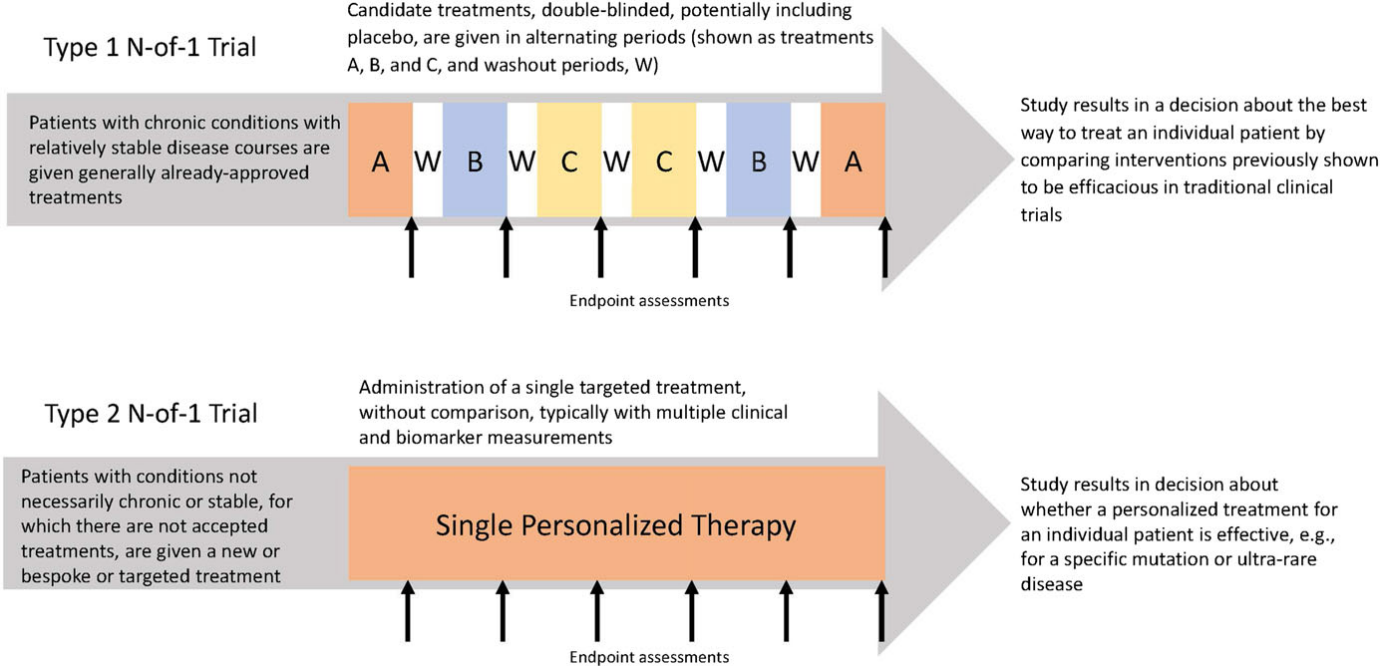
Function and structure of type 1 and type 2 N-of-1 trials.

There are two types of N-of-1 trials [7], both potentially relevant to the generation of RWD/RWE (Fig. 1). Most implemented are Type 1 trials, used to determine the best treatment for chronic conditions with relatively slow progression, which allows for legitimate sequential comparisons of candidate treatments. Rapidly progressive conditions are less suitable for this kind of N-of-1 approach, as the condition’s clinical trajectory could undermine sequential treatment comparisons. Also, ideally, candidate treatments for N-of-1 trials should have short-term effects, there should be the ability to collect RWD using reliable and validated outcome measures, and the outcomes should be reversible after stopping candidate treatments.

For patients with suitable conditions, during each treatment period, data on symptoms, signs, and/or markers of disease activity are collected by the patient, clinicians, and others involved in the patient’s care. Examples have included, in fibromyalgia, measures of pain, sleep patterns, and other symptoms [8]; in heart failure, symptoms, signs, functional assessments, and biomarkers [9]; in asthma, symptoms and pulmonary function test results [10]; and in diabetes, blood glucose levels and adverse effects [11]. N-of-1 trials have been used in pediatric conditions, in which, besides collecting data from patients and clinicians, data often are collected by parents, teachers, and therapists [12]. In all these instances, N-of-1 trials provide RWD/RWE indicating what works best for a given patient, with the advantage of treatment comparisons being done with patients serving as their own controls [4].

In addition to collecting RWD and identifying the best treatment for individuals, N-of-1 trials also can provide generalizable RWE relevant to wider populations. Data from multiple N-of-1 trials that use the same treatments and protocols can be combined as a multi-crossover design trial to provide group data [13–15]. This allows individuals to generate RWE for their personalized care and also to contribute to aggregated, generalizable RWE about the comparative effectiveness of tested treatments. However, despite this possibility, the use of N-of-1 to gather RWD and to build RWE have not been fully realized in clinical practice.

Type 2 N-of-1 trials are a more recent development, generally testing only one rather than multiple treatments. These trials typically are directed at rare or ultra-rare conditions such as a devastating disease about which there may be pathophysiologic understanding but no proven therapy. In such cases, there may be only one potentially promising treatment, and so there may be only one cycle of the candidate treatment rather than multiple treatment periods. Because sequential treatments are not being compared, it is not as important to focus only on slowly progressive conditions as it is in Type 1 trials. This single-cycle, pre-post-design is more susceptible to changes in the underlying condition than the repeated crossovers of a Type 1 N-of-1 trial but may be justified if compelling clinical and/or biomarker outcomes are achieved. This was illustrated when a treatment targeted at a physiologic defect related to a cystic fibrosis genetic variant was studied in a series of Type 2 N-of-1 trials; data collected included change in mucosal ion transport, physiologic function, and propensity for infections [16]. Other examples have tested the impact of individualized genetic drug products, such as antisense oligonucleotide products [17] and cell-based treatments [18]. As with Type 1 trials, these trials allow discernment about whether a treatment is effective for a given patient and, by using the same protocol with other patients and aggregating the data, they can inform the treatment of the condition more widely. While Type 2 trials are currently applicable to many fewer patients than Type 1 trials, with the growth of personalized molecular and cellular treatments, Type 2 N-of-1 trials are likely to find increasing use. This is reflected in recent FDA draft guidance on the use of such trials in individualized drug development [19].

Most N-of-1 trials have been done as single research projects, each facing similar challenges and barriers related to the methodology. These include the need for rigorous design, infrastructure for random allocation and blinding, procedures for washout between treatments, rescue if treatment leads to important clinical deterioration, handling treatment costs, considering ethical issues, getting institutional review board (IRB) approval, and many others. Also, when incorporated into routine patient care, we can expect tension between the needs of clinical practice versus the needs of clinical research and equipoise among candidate treatments [20–21]. We are addressing these and other challenges in the framework of the Tufts Medicine patient-centered learning healthcare system. Our objective is to create a generally available, sustained, broadly accepted infrastructure that supports standard N-of-1 procedures, processes, and practices. We are looking to reduce organizational barriers and implementation costs and to respond to patient, clinician, organizational, and payor stakeholders to facilitate embedding N-of-1 trials in clinical practice across the Tufts Medicine healthcare system. This includes capacities for both Type 1 and Type 2 N-of-1 trials to generate RWD/RWE for individual patients and for wider use.

To create a model for the use of N-of-1 trials that will provide value to patients and stakeholders, we are assembling infrastructure, processes, and systems based on input from all relevant stakeholders. We believe this is necessary for N-of-1 trials to be sustainable for broad use in the healthcare ecosystem. The steps we now are undertaking are as follows:

### Convene and Conduct Ongoing in-depth Discussions with Stakeholders

Preparatory to this project, we assembled a multi-stakeholder group to inform the development of methods, processes, standards, and platforms that would support wide availability and value of N-of-1 trials. Since December 2021, we have held monthly meetings with patients, clinicians, the health system, payors, pharmaceutical manufacturers, ethics experts, regulators, and others. The multi-stakeholder group meetings are structured to briefly share progress updates before focusing on questions related to study design, protocol development, regulatory approach, and clinician and patient engagement. The diversity of expertise and perspective represented in the stakeholder meetings has led to insights such as: working within existing payment structures to embed N-of-1 trials; preparing the local Institutional Review Board (IRB) to work with templates that will allow streamlining of N-of-1 trials; considering clinician support prior to N-of-1 recruitment, including structured training; and providing clear and actionable plain language text and visuals to N-of-1 participants.

### Create N-of-1 Trial Capacity in Our Clinical and Translational Research Center (CTRC) and Disseminate it Across the Health System, Coordinated by the CTRC

We are developing N-of-1 trial operational capacity in our CTRC for use across Tufts Medicine. This capacity is being centrally coordinated and includes a pool of clinical research coordinators that are able to support both on-site and remote consenting and data collection.

To facilitate incorporating N-of-1 trials as a “routine” part of clinical practice, the CTRC clinical research coordinators will be mobile, present in specialty clinics, and easily accessible by clinical staff to initiate participant eligibility determination and enrollment. It is anticipated that most trials will be done in the usual clinical sites for patients’ care, for example, specialty clinics or practices, with the central coordination supporting this with master or template protocols and materials, software for data collection, incorporation of trial information and outcome measures into our electronic health record, and assistance in analysis. Also, there will be coordination of practices, data acquisition, and data analysis for trials using the same protocols for the same conditions to allow aggregation of individual N-of-1 trial results to provide more generalizable evidence.

### Develop a Master N-of-1 Protocol and Manuals of Processes, Procedures, and Operations

The Master N-of-1 protocol is the overarching protocol that describes trial governance generic to all comparison protocols. Covered topics include (a) Participant Eligibility and Identification Process; (b) Participant Information and Consent Steps for Registration; (c) Generic objectives, endpoints, and endpoint justification, including Health-Related Quality of Life (HRQoL); (d) Process for Randomization and Assignment to Treatment Sequence; (e) Regulatory, Ethical, and Overall Study Oversight Considerations; (f) Key Roles and Study Governance; (g) Statistical Considerations for Individual and Aggregate Analyses; (h) Safety Oversight and Monitoring; (i) Participant Discontinuation or Withdrawal from study; (j) Data Handling and Record Keeping. While the N-of-1 (Type 1) trial design is aligned with routine care in the prescribing and administration of FDA-approved therapeutics, the N-of-1 design incorporates the rigorous measure of physician and participant self-report of condition status. In routine care, physician assessment may or may not be based using validated measures. The N-of-1 Master Protocol compels clinical assessments and participant self-report by condition-specific, validated instruments measuring outcomes such as disease activity, symptom intensity, and HRQoL. These measures are administered at baseline and at pre-determined timepoints during the study. Table 1 depicts a prototype of these objectives, endpoints, and endpoint justification as included in an N-of-1 Master Protocol. Finally, we are creating operating manuals to standardize, support, and continually improve our N-of-1 trials and to assist dissemination. This includes working closely with IRB colleagues, pharmacy colleagues, and experts in dissemination and implementation.

**Table 1.** Objectives, endpoints, and endpoint justification as included in an N-of-1 master protocol

Objectives	Endpoints	Justification for endpoints
Primary		
Determine the effect of sequential FDA-approved therapeutic agents compared to placebo on disease activity and patient-reported symptoms.	Clinical assessments, using condition-specific validated instruments measuring disease activity and symptom intensity will be administered at baseline and at pre-determined timepoints during the study as outlined in each Comparison Protocol.	A validated condition-specific questionnaire using standardized wording and response options applied to the symptoms of concerns of greatest interest to the patient. To reflect the patient’s true decision-making state, each outcome will be measured separately and reported as a measure of the treatment effectiveness. Objective condition-specific clinical assessments will measure response/improvement in disease activity.
Secondary		
Determine the effectiveness of sequential FDA-approved therapeutic agents as compared to placebo on health-related quality of life (HRQOL).	Change-from-baseline in HRQOL related to condition-specific symptoms, treatment side effects on physical health and well-being. Both participant and physician assessment will be measured.	Condition-specific symptoms and treatment side effects on physical health and well-being as perceived by the individual participant are integral to patient-centered care and provides the foundation for N-of-1 real-world evidence collection. Both participant and physician assessment will be measured and compared.
Tertiary		
Safety	Safety Endpoints will be determined by Number (and proportion) of patients reporting treatment-emergent adverse events (TEAEs) (by MedDRA system organ class and preferred term).	While all interventions apply FDA-approved therapeutics for the specific condition under study, ongoing assessment and reporting of expected and unexpected adverse events contribute to real-world evidence.

### Initiate a Program of N-of-1 Trials

The first pilot trials are being launched based on clinical requirements, opportunities for patient benefit, stakeholder input, and to generate varied experience with the approach. Inherent to the N-of-1 trial is patient-physician shared decision-making, including feedback of outcome data to individual participants, and thus the sequential treatment period results will be presented as simple, easily understandable graphs. Our first clinical examples are in severe asthma and newly diagnosed rheumatoid arthritis, both of which include a wide span of approaches to disease-modifying pharmacologic treatment, and thus are suitable for N-of-1 Type 1 trials. Both conditions are chronic with symptom severity clusters that can be assessed using established instruments that include both subjective and objective measures. Numerous pharmacologic treatment options are available, varying in mechanism of action, mode of administration, adverse event probability, severity, and cost. Clinical practice treatment guidelines may be available, but head-to-head randomized evidence is scarce for the relative effectiveness between the candidate treatments evaluated in the proposed N-of-1 design. Conducting N-of-1 trials will inform shared decision-making between each individual patient and their physician toward identifying the optimal treatment for them. Moreover, the aggregation of the series of N-of-1 trial will inform more generally the relative effectiveness of these drugs.

### Initiate Additional N-of-1 Trials

Additional trials will be initiated based on the same precepts as for the first trials, looking for diversity of conditions, patients, settings, and financial arrangements. It is our near-term objective that practitioners and patients across Tufts Medicine, and perhaps outside sponsors, be able to request support for execution of N-of-1 trials that would be helpful to them. We look forward to supporting a wide variety of such trials and providing this as an available feature of healthcare in our health system. The criteria for supporting such trials, whether Type 1 or Type 2, would depend on the appropriateness of fit to the N-of-1 approach, our capacity to support such a trial, which we intend to scale up for this purpose, and the ability to incorporate the trial into the extant healthcare ecosystem.

### Continue to Review the Operation and Results of the N-of-1 Trials with Stakeholders

Continuous improvement of our processes, infrastructure, and business arrangements will be crucial to success. We have in place and will continue to develop our continuous qualitative and quantitative feedback loop from the experience in running N-of-1 trials that will drive improvements in our evolving framework.

### Work with Other Health Systems and Stakeholders to Promote Wider Implementation

This process will include wider patient representatives and clinicians, other health systems, payors, manufacturers, and others, with the objective of creating a widely applicable N-of-1 platform in the current healthcare ecosystem. Discussions and active collaboration are now underway for others to adopt our approach and learnings.

Steps 1-5 now are underway in our CTRC and Tufts Medicine specialty clinics as pilots and will be provided as a service across the health system. This includes creating and testing systems for identifying potential candidates for N-of-1 trials and systems for the efficient and high-fidelity collection of RWD. Evolution and dissemination to other healthcare systems (steps 6 and 7) will follow, with continuing and wider stakeholder engagement. Thereby, we hope to be able to provide both high-quality real-world care and RWE to advance care of individual patients and for general application in many conditions and for wide populations of patients.

## Discussion

The use of RWD/RWE has been introduced into regulatory decisions and there is growing interest in using observational RWD to support decision-making. Whether observational RWD/RWE are well-suited for clinical and regulatory decision-making is debated because of the well-known limitations associated with estimating treatment effects from observational non-randomized studies [22]. N-of-1 trials can generate patient-centered evidence in the context of real-world care with the rigor of randomized trial methods.

We believe that this approach offers an excellent use of RWD/RWE and illustrates the use of individual patient data collection to support care and to assess efficacy for larger populations. Moreover, by incorporating N-of-1 trials into real-world care, patients and the public could become more familiar and appreciative of the need to carefully evaluate treatments.

We note that this approach, which applies clinical trial methods to individual patient care, is adjacent to data collection as done in the context of the Compassionate Use of medications that are not yet approved for a given condition. Increasingly, RWD are collected for such cases, often rare or dire conditions, and yet there are intrinsic biases in this approach [23,24]. The selection of patients for such compassionate use will potentially depend on the patient’s access to specialized care. Additionally, data collection will depend on the circumstances of the patient and whether this compassionate care access is renewed (e.g., annually), which will be confounded by whether the medication is perceived to have helped and other circumstances and by whether the patient has survived. Bringing the more rigorous processes and data collection of a formal Type 2 N-of-1 trial could allow access to the treatment while also more fully evaluating its effects. This also would allow compiling data from similar patients worldwide who are using that treatment for a rare or dire condition. This potentially could lead to regulatory approval for a treatment that otherwise might not have gained the attention and resources needed to mount a conventional pivotal trial for regulatory approval. This would be an example of RWD/RWE generated by real-world care that could advance individual and population health.

We believe that Type 1 N-of-1 trials will most often be used to evaluate already approved drugs. The results of these trials could help clinicians, the health system, payors, pharmacy benefit managers, and pharmaceutical companies agree on a shared approach to the use (and payment for) expensive medications – basing decisions on the results of the double-blinded testing of the treatment options for the given patient. This might be particularly helpful in considering an off-label use of an approved drug in the face of interests in limiting such use. Moreover, beyond the individual patient, relevant to the populations of similar patients, the results of multiple N-of-1 trials testing off-label treatments for a given condition might be aggregated and used to apply for regulatory approval for a new indication [7].

We note that besides the uses described above, the N-of-1 approach may be particularly well-suited to learning health systems. The focus on individual patients’ best treatment and the appreciation of aggregated data across the health system could improve individual and population health. Indeed, the fact that such evidence can guide care in a health system in its ecosystem is a reason that the Tufts Medicine system uses the moniker of “patient-centered learning health system.”

## Conclusion

Ideally, the use of RWD to generate RWE should lead to improvement of real-world data-driven care. We believe that N-of-1 trials can have multiple levels of impact on care – on individual patients, on populations with certain conditions, and in the execution of care in our healthcare ecosystem. The involvement of individual patients, and of stakeholders, in the execution and use of N-of-1 trial evidence also should familiarize those in healthcare to the benefits of systematic study of treatments, bringing research closer to their experience, hopefully engendering greater appreciation of this approach and the collection of RWD. We are testing this at Tufts Medicine and believe it merits evaluation by other clinicians and health systems as well.
